# Excision of HIV-1 Proviral DNA by Recombinant Cell Permeable Tre-Recombinase

**DOI:** 10.1371/journal.pone.0031576

**Published:** 2012-02-13

**Authors:** Lakshmikanth Mariyanna, Poornima Priyadarshini, Helga Hofmann-Sieber, Marcel Krepstakies, Nicole Walz, Adam Grundhoff, Frank Buchholz, Eberhard Hildt, Joachim Hauber

**Affiliations:** 1 Heinrich Pette Institute – Leibniz Institute for Experimental Virology, Hamburg, Germany; 2 Department of Medical Systems Biology, University Hospital and Medical Faculty Carl Gustav Carus, University of Technology Dresden, Dresden, Germany; 3 Max Planck Institute for Molecular Cell Biology and Genetics, Dresden, Germany; 4 Department of Virology, Paul Ehrlich Institute, Langen, Germany; University Hospital Zurich, Switzerland

## Abstract

Over the previous years, comprehensive studies on antiretroviral drugs resulted in the successful introduction of highly active antiretroviral therapy (HAART) into clinical practice for treatment of HIV/AIDS. However, there is still need for new therapeutic approaches, since HAART cannot eradicate HIV-1 from the infected organism and, unfortunately, can be associated with long-term toxicity and the development of drug resistance. In contrast, novel gene therapy strategies may have the potential to reverse the infection by eradicating HIV-1. For example, expression of long terminal repeat (LTR)-specific recombinase (Tre-recombinase) has been shown to result in chromosomal excision of proviral DNA and, in consequence, in the eradication of HIV-1 from infected cell cultures. However, the delivery of Tre-recombinase currently depends on the genetic manipulation of target cells, a process that is complicating such therapeutic approaches and, thus, might be undesirable in a clinical setting. In this report we demonstrate that *E.coli* expressed Tre-recombinases, tagged either with the protein transduction domain (PTD) from the HIV-1 Tat *trans*-activator or the translocation motif (TLM) of the Hepatitis B virus PreS2 protein, were able to translocate efficiently into cells and showed significant recombination activity on HIV-1 LTR sequences. Tre activity was observed using episomal and stable integrated reporter constructs in transfected HeLa cells. Furthermore, the TLM-tagged enzyme was able to excise the full-length proviral DNA from chromosomal integration sites of HIV-1-infected HeLa and CEM-SS cells. The presented data confirm Tre-recombinase activity on integrated HIV-1 and provide the basis for the non-genetic transient application of engineered recombinases, which may be a valuable component of future HIV eradication strategies.

## Introduction

Viral infectious diseases like HIV/AIDS affect millions of people worldwide and have been a global health concern for many years. Since the beginning of the HIV epidemic the treatment methods have been significantly evolved, resulting in the present highly active antiretroviral therapy (HAART), a combination of various antiretroviral drugs [1], [2]. These approaches have been effective in controlling viral replication and extending the life-span of infected individuals. HAART, which is targeting the viral proteins reverse transcriptase [3]–[5], protease [6], integrase [7], or virus entry [8]–[10] has transformed HIV-1 infection into a chronic illness [11]. Despite the clinical benefits of HAART the prospect of life-long anti-retroviral treatment poses significant problems such as the occurrence of drug-related toxicities [12]. Concomitantly, HAART has considerable impact on the daily life through strict scheduling requirements [2]. Withdrawal of HAART and suboptimal drug adherence frequently results in emergence of drug-resistant viruses [13], [14]. Hence, there is a pressing need for new strategies to improve and extend current therapy options, preferable by regimes that not only block virus replication but are also able to eliminate HIV-1 from persistent viral reservoirs [15]. An attractive therapy approach employs a LTR-specific Tre-recombinase, which has been demonstrated to be a promising tool for excision of the HIV-1 provirus from infected cell cultures by recombining LTR sequences [16], [17]. To date, however, the delivery of Tre-recombinase depends on gene therapy approaches, in which patient cells are modified to contain advanced Tre-expressing vector systems [18]–[20].

A key obstacle to the development of new antiviral agents is their efficient and safe delivery into infected cells *in vivo* without causing adverse side effects in the relevant target cells [21]. Many different technologies have been reported to improve the cellular uptake of therapeutic macromolecules such as proteins, nucleic acids or peptides [22]–[24]. Currently the most popular and efficient technique for achieving the intracellular access of such molecules exploits so-called protein transduction domains (PTD) or cell penetrating peptides (CPP) from different sources [25]. PTD/CPP (here generally referred to as PTD) based strategies were highly successful in the delivery of various genes and proteins [21], [26], [27], including site-specific recombinases [28]–[30]. PTD have been employed in preclinical models of human diseases such as cancer, psoriasis, and stroke [31]–[34]. However, none of these approaches have yet advanced into the clinic. The most studied and applied PTD are represented by peptides derived from the basic domain of the HIV-1 Tat *trans*-activator, the homeodomain of *Drosophila antennapedia* (Antp) and the HSV VP22 transcription factor [35]–[37]. Furthermore, a powerful novel cell permeable translocation motif (TLM), derived from the PreS2 surface antigens of Hepatitis B virus (HBV), has been previously reported [38]. The TLM, which is a 12 amino-acid amphipathic α-helical peptide, mediates the energy and receptor-independent transfer of peptides, nucleic acids and proteins across plasma membranes. This process apparently occurs without affecting the integrity of the cell or interfering with intracellular signal transduction cascades [38], [39].

Here we describe the PTD-mediated delivery of biologically active Tre-recombinase into human cells. Recombination of HIV-1 LTR-specific sequences was determined by using transient and stably integrated HIV-1 reporter constructs. The obtained results suggest that the direct delivery of cell permeable Tre-recombinases (CPTR) into HIV-infected cells may be a valuable and safe component of future antiretroviral strategies that aim at virus eradication.

## Results

### Cloning, over expression and purification of Tre-recombinases in *E.coli*

**Figure 1:**
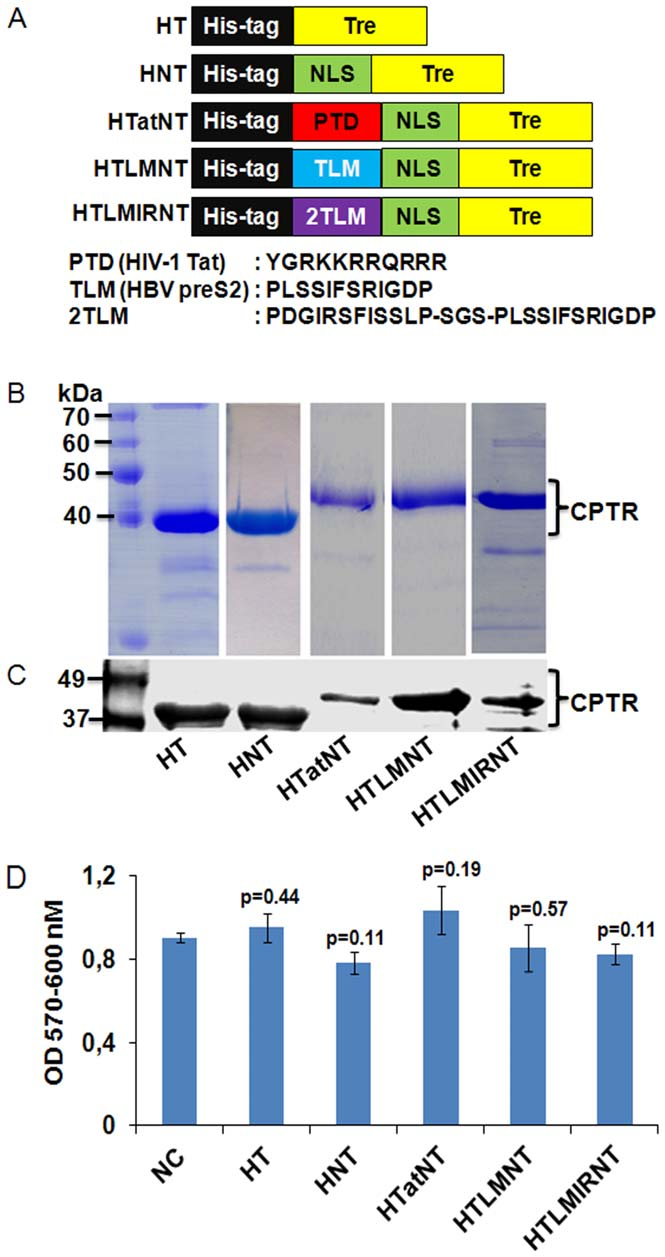
Over expression of cell permeable Tre recombinases. (A) Schematic representation of the Tre-recombinase constructs used in this study. (B) Coomassie stained SDS-PAGE (12%) of the *E. coli* expressed and purified proteins. (C) Western blot analysis of Tre-recombinase proteins using anti-Tre polyclonal antibodies. (D) Analysis of potential Tre-induced cellular toxicities. HeLa cells were exposed for 48 h to 1 µM of the indicated recombinant fusion proteins. Cellular metabolic activity was subsequently tested by alamarBlue assay. NC, negative control experiment in which Tre protein was omitted. Numbers over each bar indicates the p values calculated by paired t-test.

Five Tre-recombinase expression constructs were generated in order to analyze the cell permeability and activity of Tre on its HIV-1 LTR DNA substrate, a 34-bp asymmetric sequence from the primary subtype A HIV-1 strain TZB0003 termed LoxLTR [16], [40]. As depicted in Figure 1A, the expression construct HT represents a protein, in which an N-terminal hexa histidine tag (His-tag) is fused to Tre, which can be cleaved off by using thrombin protease. The HNT protein contains the canonical nuclear localization signal (NLS) from the SV40 large T antigen [41] inserted between the His-tag and Tre-recombinase. The HTatNT and HTLMNT constructs were generated by inserting either the PTD from HIV-1 Tat or the HBV preS2 TLM between the His-tag and NLS-Tre. Finally, the HTLMIRNT construct was generated by inserting the TLM sequence as an inverted repeat (2TLM).

The HT, HNT, HTLMIRNT genes were inserted into the bacterial expression plasmid backbone pET28b (Novagen), while the HTatNT and HTLMNT constructs were ligated into the vector pTrcC (Invitrogen) as described in the methods section. All five fusion proteins were expressed in the *E.coli* Rosetta strain (Novagen) and the respective proteins were purified using Ni-NTA metal-ion affinity and HiLoad superdex S200 gel filtration chromatography (GE Healthcare). While the solubility and the yield of each protein was different in each culture, the purified proteins displayed the expected molecular mass (∼40.0–45.0 kDa) when analyzed by SDS-PAGE (Figure 1B) and reacted with anti-Tre polyclonal antibodies in Western blot experiments (Figure 1C).

Prior to the following cell permeability studies, it was important to determine potential cellular toxicities of the *E. coli* expressed recombinant CPTR. Hence, HeLa cells were incubated with the purified proteins for 48 h (the entire time-span of the subsequent experiments) and their effect on cellular metabolic activity at the highest concentration used (1 µM) was determined by alamarBlue (Serotec) assay. As shown in the Figure 1D, no Tre-induced undesired effects on cellular metabolism were detected in these experiments.

### Cell permeability of recombinant Tre-recombinases

**Figure 2:**
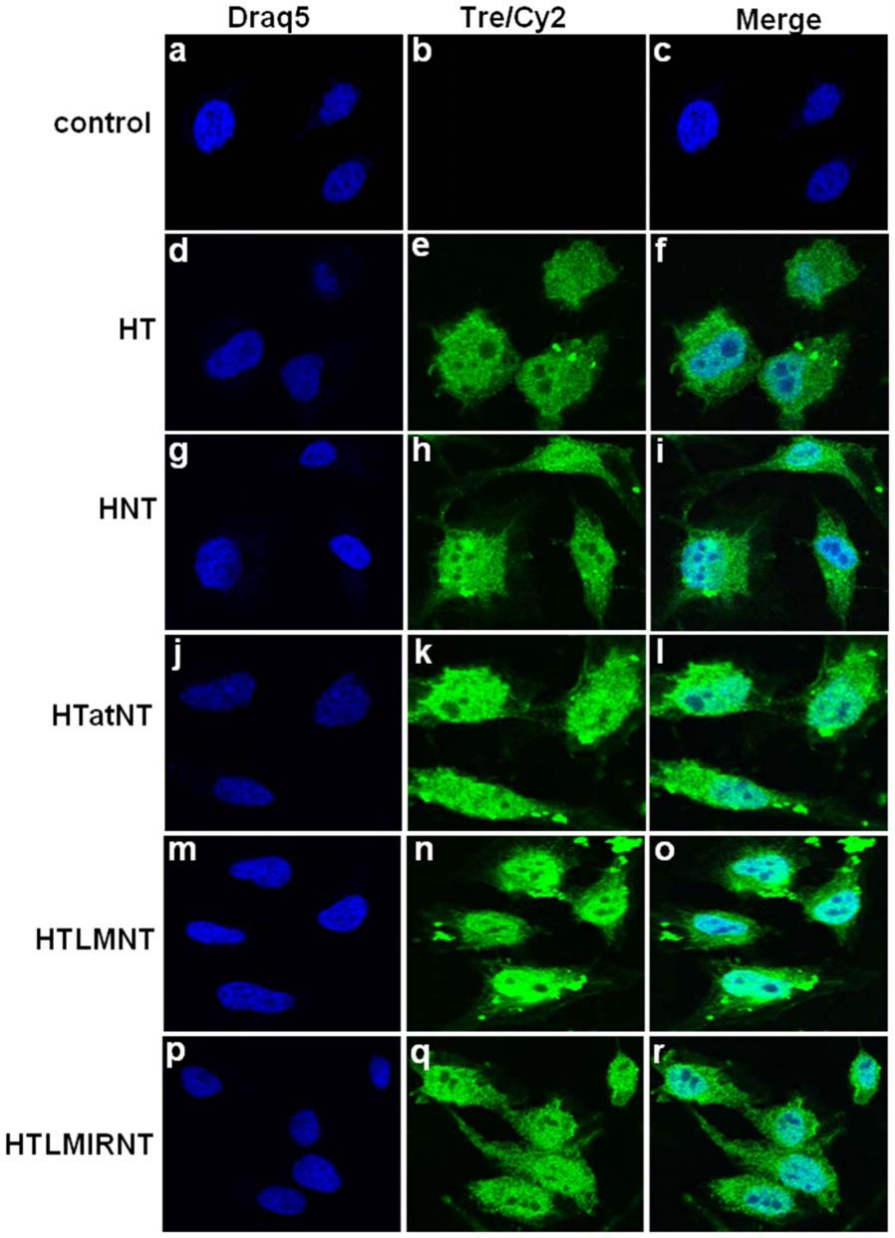
Subcellular localization of cell permeable Tre in transduced HeLa cells. Cellular uptake and localization of the indicated recombinant fusion proteins were studied by confocal laser scanning microscopy in HeLa cells. HeLa cells were exposed for 5 h to 1 µM of the various Tre-recombinases. Subsequently, the respective cell cultures were washed twice with PBS and PBS containing 0.5 mg per ml heparin for 5 min each. Nuclei were stained with Draq5 (blue label), Tre-recombinases (green label) with a primary polyclonal anti-Tre and secondary Cy2-labeled antibodies.

Confocal laser scanning microscopy using primary polyclonal antibodies directed against Tre and FITC-conjugated secondary antibodies was performed in order to determine the cellular localization of the various recombinant Tre proteins. The respective images show Tre-specific signals in both the cytoplasm as well as in the nucleus for all five proteins tested (Figure 2), suggesting that all of the recombinant Tre-recombinases were able to internalize into HeLa cells. Surprisingly, the Tre protein lacking NLS and PTD (i.e. HT; Figure 2, panel d–f), as well as the NLS-Tre fusion (i.e. HNT; Figure 2, panel g–i) was apparently also able to internalize. Interestingly, this behavior was previously observed in case of the parental Cre recombinase protein, which has been shown to possess an intrinsic property to transduce into mammalian cells without the support of any PTD sequence [42], [43]. Since Tre-recombinase is a protein engineered from Cre [16], its obviously intrinsic property of internalization can be explained by this Cre-related property. However, the intensities of the signals for HTatNT, HTLMNT and HTLMIRNT (Figure 2, panel j–l, panel m–o and panel p–r, respectively) were more prominent as compared to the proteins lacking a PTD (i.e. HT and HNT; panel d–f and panel g–i, respectively), indicating enhanced protein transduction efficiency. The experimental settings for confocal microscopy were identical for all panels shown (Figure 2). Since HTatNT contains a cell penetrating peptide motif derived from the HIV-1 Tat *trans*-activator protein (Figure 1A), the potential *trans-*activation capacity of HTatNT was analyzed in a control experiment (Figure S1 and File S1) demonstrating, that HTatNT cannot activate the HIV-1 LTR promoter. Thus, it is highly unlikely that HTatNT treatment of HIV-1 infected cells will activate the integrated provirus.

### Analysis of Tre activity

**Figure 3:**
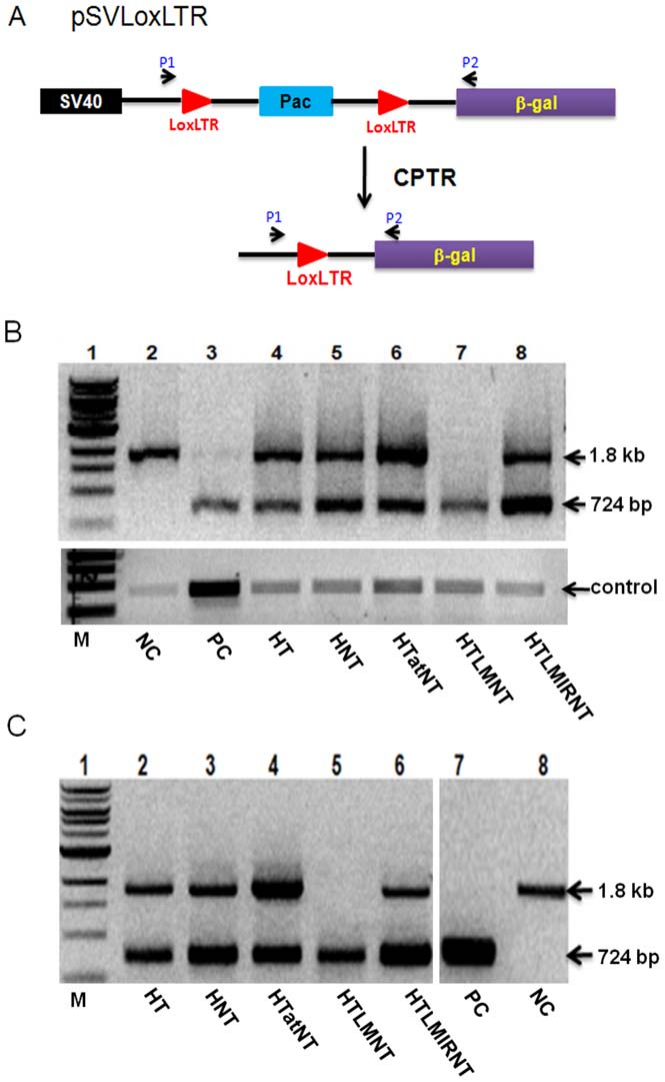
Analysis of Tre activity in HeLa cells. (A) Schematic diagram of the pSVLoxLTR reporter construct. The LoxLTR Tre target sequences and the P1 and P2 PCR primer sites, used for the monitoring of Tre-mediated recombination, are indicated. Recombination results in the de novo detection of a 724 bp PCR product. SV40 indicates the SV40 promoter. (B) HeLa cells were transiently transfected with pSVLoxLTR reporter plasmid, exposed to the indicated recombinant Tre proteins (lane 4–8) and recombination activity was monitored by PCR. M, DNA size markers; NC, negative control reaction lacking Tre; PC, positive control reaction in which Tre was coexpressed from the contransfected p3Tre plasmid. (C) Stable LoxLTR HeLa cells were treated with 1 µM of the indicated CPTR and recombination activity was analyzed as before.

The activities of the recombinant PTD-Tre-recombinases were first examined in a transient reporter assay. In Figure 3A the schematic diagram of the pSVLoxLTR reporter construct is depicted. The pSVLoxLTR plasmid contains a puromycin resistance gene (pac), which is flanked by Tre-recombinase target sites (LoxLTR) followed by sequences encoding β-galactosidase (β-gal). Thus, Tre-mediated recombination of LoxLTR results in pac excision, which can be detected by PCR using the indicated primer binding sites (denoted P1 and P2 in Figure 3A). In fact, a 1.8 kb PCR product indicates unrecombined pSVLoxLTR plasmid, while a 724 bp fragment is indicative for the successful recombination of its LoxLTR sites.

HeLa cells were transiently transfected with the pSVLoxLTR reporter construct and protein transduction was performed by incubating the transfected cells with 1 µM of the various purified Tre proteins for 5 h. The level of recombination was determined 48 hours post protein transduction by PCR using whole genomic DNA. No recombination was detected in a negative control experiment lacking Tre-recombinase (Figure 3B, upper panel lane 2). In contrast, Tre-mediated recombination could be easily verified in a positive control experiment in which HeLa cells were cotransfected with the p3Tre expression plasmid (Figure 3B, upper panel lane 3). Likewise, cells treated with all CPTR resulted in the amplification of a 724 bp fragment, demonstrating that protein transduction is an efficient way to deliver Tre activity into cells (Figure 3B). For transfection efficiency control, all DNA samples were also subjected to PCR control of vector derived *amp*-specific sequences (Figure 3B, lower panel). Please note, that the observed enhanced signal in case of the positive control experiment (Figure 3B, lower panel, lane 3) resulted from the coamplification of the pSVloxLTR- and p3Tre-derived sequences. However, we were unable to detect the unrecombined PCR product in the positive control culture (Figure 3B, lane 2) as well as in HTLMNT (Figure 3B, lane 7) treated cells, indicating high recombination efficiency. The remaining Tre variants displayed both unrecombined and recombined PCR products, (Figure 3B, upper panel lane 4–6 and lane 8). These results were also confirmed by using a transient Tat *trans*-activation assay (see Figure S2 and File S1). Taken together, these data showed that *E.coli* expressed recombinant CPTR are taken up by HeLa cells and are able to recombine episomal LoxLTR sequences.

The essential function of a CPTR, however, is its recombination activity at the genomic level, which is prerequisite to the application as a tool for the removal of proviral DNA from HIV-1 infected cells. Hence, to determine the function of Tre-recombinases at the chromosomal level we next employed HeLa cells in which the pSVLoxLTR reporter construct was stably integrated into the genome. After protein transduction of these cells, genomic DNA was again isolated and analyzed by PCR as before. As shown and in agreement with the previous transient experiments (Figure 3B), all recombinases were able to recombine genomic LoxLTR sequences to some extent (Figure 3C). Once again, we were unable to detect the unrecombined PCR product in HTLMNT fusion protein treated cells, which may be due to highest recombination efficiency among all proteins tested (Figure 3C, lane 5). These data indicated that the recombinant CPTR proteins are able to enter target cells and can also recombine LoxLTR sequences in a genomic context.

### Half-life determination of selected CPTR

**Figure 4:**
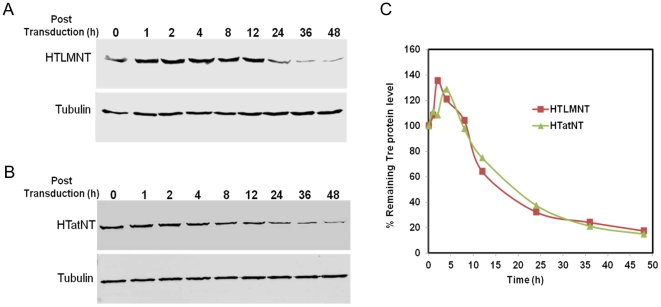
Protein stability of selected CPTR in mammalian cells. Total cellular lysates were prepared at indicated time points from (A) HTLMNT and (B) HTatNT transduced and HIV-1 infected CEM-SS cells. Tre-recombinases were detected by Western analysis using anti-Tre polyclonal antibodies (upper panel). Equal sample loading was verified by detection of tubulin (lower panel). (C) Relative intensity of CPTR proteins at indicated time points.

During the preparation of the various recombinant CPTR we noted that particularly the HNT and HTLMIRNT proteins were highly labile and precipitated quickly (not shown). We therefore wanted to investigate the half-life of the HTLMNT recombinase and of the HTatNT protein in more detail, particularly after their internalization into mammalian cells. CEM-SS T cells were exposed to 1 µM of HTatNT or HTLMNT protein and cell lysates were prepared at various time points (indicated in Figure 4A and 4B) after protein transduction and Tre stability was analyzed by Western blotting. The HTLMNT and HTatNT proteins were detected over the entire time period (Figure 4A and 4B) and degradation occurred with comparable kinetics, resulting in the degradation of ∼90% of input protein at the 48 h time point of incubation (Figure 4C).

### Interaction of CPTR and the LoxLTR target site in living cells

**Figure 5:**
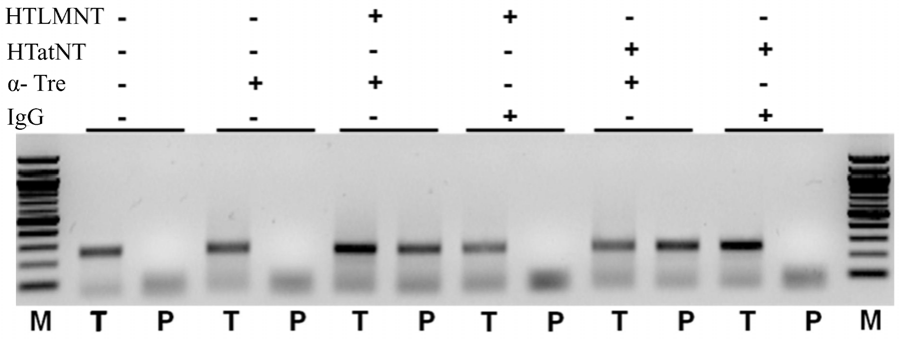
Interaction of CPTR with LoxLTR sites in living cells. (A) Schematic representation of human genomic DNA containing HIV-1 provirus with flanking LTR. The Tre recombinase binding sequences, LoxLTR, shown in black box and red arrows indicates the primers used for PCR analysis. (B) ChIP assay of extracts derived from CPTR transduced and HIV-infected CEM-SS T cells. The Tre-specific LoxLTR target site was detected by PCR analysis. M, 1 kb NEB marker; T, 1∶10 diluted total input samples (positive PCR control); P, pull-down of Tre-recombinase using specific antibodies (α-Tre) or non-specific IgG. The LoxLTR-specific PCR products are shown.

Next we performed chromatin immunoprecipitation (ChIP) assays using HIV-1 infected CEM-SS T cells to directly demonstrate the binding of selected CPTR to their specific LoxLTR sequence in living cells. After transduction of these cells with HTLMNT and HTatNT proteins, cellular extracts were prepared and formaldehyde-crosslinked protein:DNA complexes were immunopreciptated using Tre-specific antibodies. Bound LoxLTR sequences were amplified by specific PCR and, for visualization, were subjected to agarose gel electrophoresis (Figure 5). Furthermore, the PCR products were confirmed by DNA sequencing (not shown). The respective results demonstrated that both CPTR were clearly able to recognize and bind to chromosomally integrated LoxLTR sequences in living cells.

### Expression analysis using whole human genome microarrays

To investigate whether treatment with CPTR may significantly alter the cellular expression profile, we performed a transcriptome analysis using whole human genome microarrays (see supplementary methods for details). The complete dataset has been deposited in NCBI's Gene Expression Omnibus (GEO) database, accession number GSE32643 (http://www.ncbi.nlm.nih.gov/geo/query/acc.cgi?acc=GSE32643). As shown in Figure S3 (see also File S1), at 48 hours post treatment only a few genes showed differential expression levels when compared to HTLMNT untreated CEM-SS cells. Overall, we observed 17 and 25 genes which were more than twofold up- or downregulated, respectively (see Tables S1 and S2). Out of these, only 8 genes showed a more than 2.5-fold expression change, and only a single gene (HMGCS1, see Table S2) was regulated more than threefold, indicating that the expression profile of CPTR-treated cells does indeed not differ substantially from the untreated control cells.

### Recombination of the full-length HIV-1 proviral genome

**Figure 6:**
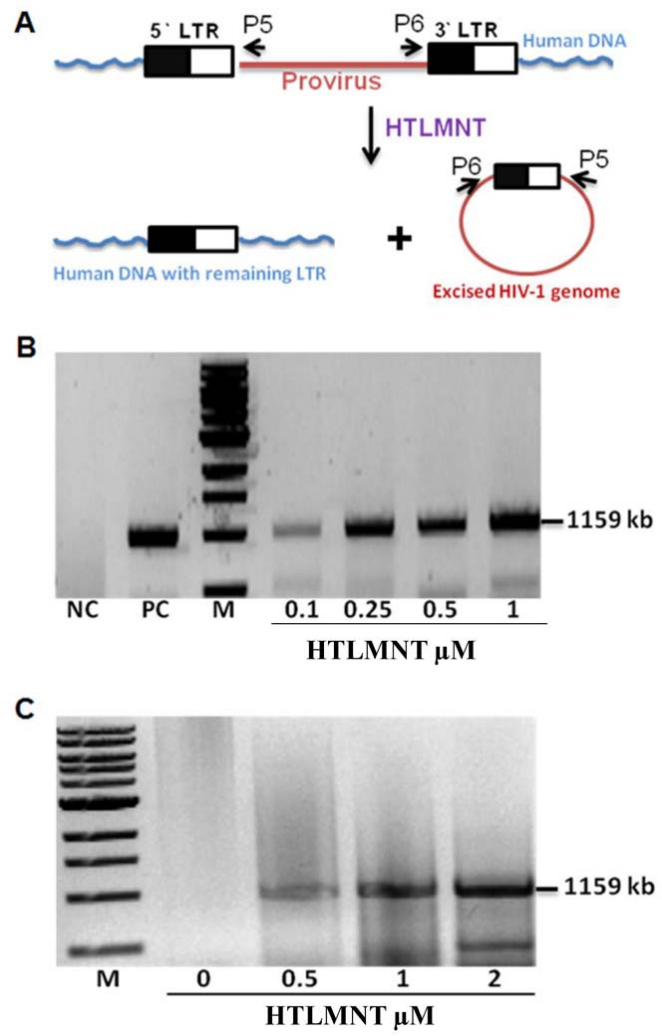
Excision of integrated HIV-1 proviral genomes. (A) Depiction of the integrated proviral DNA and the products originating from Tre-mediated LoxLTR recombination. P1 and P2 denote PCR primer binding sites used for the detection of the excised circular recombination product. HIV-1 infected HeLa (B) and CEM-SS (C) cells were exposed to the indicated concentrations of recombinant HTLMNT protein. At 48 h post protein transduction genomic DNA was isolated and subjected to PCR. The recombination product is represented by the amplification of a 1.1 kB DNA fragment. NC, negative control in which HTLMNT was omitted; PC, positive control in which Tre was coexpressed from the p3Tre expression vector; M, DNA size markers.

The results obtained at this point demonstrated that CPTR proteins were able to recognize and recombine HIV-1 LoxLTR sequences. In particular, we were unable to detect any unrecombined PCR product in HTLMNT-treated cells, both at the episomal and chromosomal level. Moreover, the HTLMNT protein was more soluble than the other CPTR tested (data not shown). Hence, we next wanted to determine the potential of the HTLMNT protein to excise full-length HIV-1 genomes from their chromosomal integration sites. First, we generated HeLa and CEM-SS cells that were infected with pseudotyped HIV-1 and therefore contain integrated full-length HIV-1 proviral DNA (Figure 6A). We then tested the ability of transduced HTLMNT to excise the provirus from the genome. Tre-mediated recombination of the respective LoxLTR sequences leaves a single LTR at the respective chromosomal integration site and produces a circular 1-LTR recombination product, which is ultimately degraded by cellular nucleases. Indeed, by using HIV-1 gene-specific oligonucleotide pairs (denoted P3 and P4 in Figure 6A), the excised HIV-1 genome was specifically detected by PCR, thereby indicating Tre-mediated recombination. Furthermore, after exposure of these HIV-1 infected cells to increasing amounts of HTLMNT protein, recombination of LoxLTR occurred in a dose-dependent manner and was even observed at the lowest concentration tested, both in HeLa cells (Figure 6B) and CEM-SS T cells (Figure 6C). Moreover, DNA sequencing of the respective PCR products resulted in the detection of the expected HIV-1-derived sequences, including the Tre-specific LoxLTR target site (data not shown). We therefore conclude that CPTR are useful for the excision of integrated HIV-1 proviral DNA from the genome of infected cells and may therefore present important components of advanced future antiretroviral therapies.

## Discussion

In the past decade there has been tremendous progress in the development of drug delivery techniques and the application of PTD seems more accessible than ever, although this technique has so far not been introduced into the clinic. The main advantage of using these systems is their ability to deliver bioactive molecules, including genes, siRNA, oligonucleotides, peptide nucleic acids (PNA), proteins, peptides and liposomes into all types of cells *in vitro* and, furthermore, into various organs *in vivo* [21], [26], [27]. Thus, PTD's intrinsic internalization properties have been exploited in many new therapeutic applications [25] and may therefore play an increasingly important role in future disease treatments [27]. In fact, PTD fused proteins are already widely used for delivering anticancer agents (reviewed in [44]). For example, the HIV-1 Tat peptide fused to Bcl-xL and pyrimidine dimer glycolase proteins was used in ischemic brain injury and skin cancer treatment, respectively [31], [45], [46]. Moreover, the PTD derived from the HSV VP22 protein was exploited for the delivery of the GAT4 transcription factor to combat myocardial injury [47].

Technically, the tagging of a PTD to the cargo protein can be easily achieved via molecular cloning and subsequent expression of the respective fusion protein [45], [47], [48]. In particular, a novel PTD or translocation motif (TLM with high transfer efficiency) has been discovered in the hepatitis B virus PreS2 protein [38], [49]. Its low immunogenicity, high spreading capacity, and its defined structure-function relationship has made this PTD an efficient and valuable tool for biomolecule delivery [32], [38], [50].

Novel experimental therapies for the treatment of HIV/AIDS focus on the eradication of HIV-1 in infected individuals, thereby potentially providing a cure for this chronic and life-threatening disease (reviewed in [51], [52]). In this respect various RNA-based technologies are currently investigated, including application of RNA aptamers, siRNA and shRNA, TAR decoys and ribozymes [53]–[57]. Clearly, these approaches efficiently suppressed virus replication and reduced viral loads for extended periods of time, but so far failed in virus eradication. In that respect, mainly two different antiviral strategies are currently investigated. The genomic disruption of the *CCR5* gene, encoding the essential cellular coreceptor of CCR5-tropic (R5) viruses, by expressing engineered zinc finger nucleases (ZFN), has been shown to result in impaired surface expression of CCR5 and, consequently, in resistance to *de novo* infection by CCR5-tropic HIV-1 [58], [59]. Another strategy, which is independent of the tropism of the virus, targets already HIV infected cells by removing the integrated proviral DNA from the host cell. This approach is based on the cellular expression of gene sequences encoding a tailored LTR-specific recombinase, which was obtained *in vitro* by directed molecular evolution technology and recognizes a primary HIV-1 subtype A isolate [16], [17]. Such a Tre-recombinase-based therapeutic approach may be accomplished by direct gene transfer of Tre encoding sequences into potential HIV-1 host cells (or respective progenitor cells). However, this gene transfer requires the use of advanced vector systems, that are frequently derived from pathogenic viruses [20], [60], [61]. Therefore, the required vector-mediated gene transfer may by itself pose some significant health risks [60], [62], [63]. It would be therefore advantageous if antivirals, such as biologically active Tre-recombinase (or engineered ZFN), could be directly and efficiently delivered into host cells.

Such a strategy could be particularly conceived in case of T cell-based therapies in which the patient's PBMC can be easily harvested by apheresis [64]. The purified T lymphocytes would then be transduced *in vitro*, potentially expanded and reinfused into the respective donor. By transducing these cells directly with cell permeable antivirals, such as for example CPTR, gene transfer procedures could be complemented or entirely avoided, an aspect that may significantly improve the safety of such advanced therapies.

The data raised in the present study suggest that CPTR, and in particular the HTLMNT variant of recombinant Tre-recombinase, is useful in such or a comparable therapy procedure. As shown, all the tested proteins purified from *E.coli* to near homogeneity using two chromatography techniques, were not toxic in HeLa cells (at a maximum concentration of 1 µM) and were efficiently internalized by cells when added to the respective cultures. Importantly, all tested recombinant Tre proteins were able to excise LoxLTR flanked regions of HIV-1, both on transiently transfected reporter plasmids as well as in the genomic context in stable reporter cell lines. Although the HT protein, which lacks any PTD and NLS, was able to cross the cellular plasma membrane, it was clearly less active as compared to PTD tagged proteins (HTatNT and HTLMNT). The HTLMNT protein displayed high solubility, stability and apparently the highest efficiency of all Tre variants tested. This might reflect the fact that in contrast to the HIV-1 Tat-derived peptide, HBV PreS2 TLM-dependent translocation occurs independent from endocytic processes [39], [65]. Rather, TLM-fused proteins translocate directly across the cellular membrane into the cytoplasm [39], a process that might help to preserve a higher enzymatic activity and a longer protein half-life. Further experiments are in progress to optimize the technology and to investigate the protein kinetics and activity in different cell lines and primary cells. However, this first report and data of cell permeable recombinant Tre activity indicates the potential of these proteins for use as a nano-surgical tool to potentially reverse HIV-1 infection and thereby curing infected cells. Thus, the application of recombinant PTD-Tre might contribute to future antiretroviral therapies of the post HAART era.

## Materials and Methods

### Generation of cell permeable Tre-recombinases

The XL-gold strain of *E. coli* (Agilent Technologies) was used for cloning, DNA sequencing and propagation of plasmids. All of the Tre-recombinase proteins were amino-terminal hexa histidine tagged (H) with a thrombin cleavage site separating the His-tag and the Tre-recombinase. The Tre-recombinase (T), without nuclear localization signal (NLS) and PTD is designated as HT and with NLS is designated HNT. The HT and HNT constructs were inserted in the pET28b vector at *Nde*I and *Xho*I sites using the following primers; forward primer for HT; 5′-CG*CATATG*TCCAACCTGCTGACC-3′, forward primer for HNT; 5′-CG*CATATG*GTGCCCAAGAAGAAGCGGA-3′ and reverse primer for both HT and HNT; 5′-CG*CTCGAG*TCAATCGCCATCTTCCAGCAG-3′. The sequences in italics correspond to *Nde*I and *Xho*I sites in the forward and reverse primers, respectively. The HIV-1 Tat PTD and HBV PreS2 TLM -tagged Tre-recombinase proteins were designated HTatNT and HTLMNT, respectively. The HTatNT and HTLMNT constructs were propagated in the pTrcHisC vector as follows. First, the order of the *Hind*III and *Xho*I restriction sites in the parental pTrcHisC vector was changed by insertion of an oligonucleotide containing the Tat-PTD sequence using primers (forward primer; 5′-GATCTCTGGCCGTAAAA AGCGCCGTCAGCGCCGCCGT AAGCTTGGTGCTTCTCATCTCGAGT-3′; and reverse primer 5′- AGCTACTC GAGATGAGAAGCACCAAGCTTACGGCGGCGCTGACGGCGCTTTTTACGGCCAGA-3′), or without the Tat-PTD-sequence (forward primer; 5′- GATCTCTAAGC TTGGTGCTTCTCATCTCGAGT-3′ and reverse primer; 5′-AGCTACTCGA GATGAGAAGCACCAAGCTTAGA-3′). The Tre sequence was subsequently inserted into the *Hind*III and *Xho*I vector sites. The construct without Tat-PTD was used to generate HTLMNT by insertion of a TLM-oligonucleotide into the *Hind*III site (forward primer 5′-AGCTACCCTTATCGTCAATCTTTTCGCGAATCGGCGATCCTG-3′; reverse primer 5′-AGCTCAGGATCGCCGATTCGCGAAAAGATTGACGATAAGGGT-3′). Tre tagged with inverted TLM sequences was designated as HTLMIRNT and was also generated in two steps. First, the TLMIR sequence, encoding the amino acid sequence NH_2_-PDGIRSFISSLP-SGS-PLSSIFSRIGDP-COOH, was PCR amplified from the vector pAS2-TLM2X [39] using the forward primer 5′-CGA*CATATG*CCTGATG GCATCCGATCG-3′ and the reverse primer 5′-CGA*GGATCC*AGGGTCCCCAATCCT CGAGAA-3′ and inserted into the *Nde*I and *BamH*I sites of pET28b. Next, the Tre- recombinase gene with NLS was fused at *BamH*I and *Hind*III sites using the forward primer 5′-CG*GGATCC*ATGGTGCCCAAGAAGAAG-3′ and the reverse primer 5′-CGC*AAGCTT*TCAGTCGCCGTCCTCCAGCAGTCT-3′. All constructs were confirmed by DNA sequencing using T7 sense and antisense primers.

The *E. coli* strain Rosetta (Novagen) was employed for protein expression. The respective genes were cloned in different vectors for expression in the various host systems depending upon their expression and solubility.

### Over expression of Tre-recombinases

For over expression the HT, HNT and HTLMIRNT encoding plasmids were transformed into *E.coli* Rosetta cells (Novagen), plated on Luria-Bertini (LB)-kan dishes (50 µg/ml kanamycin) and incubated at 37°C for 12 h. A single colony was used for inoculation of 50 ml of LB-medium containing 50 µg/ml kanamycin and grown for 12 h at 37°C (pre-inoculum). This pre-inoculum (4%) was then used to inoculate 500 ml of terrific broth (TB) containing 50 µg/ml kanamycin (450 ml TB medium +50 ml of 0.17 M KH_2_PO_4_ and 0.72 M K_2_HPO_4_) and cultured at 37°C until an O.D._600_ of 0.6 was reached. Tre-recombinase gene expression was induced by addition of 0.5 mM of IPTG for a period of 5 h at 30°C. Over expression of HTatNT and HLTMNT was carried out using the same protocol, except for using ampicillin selection (100 µg/ml) instead of kanamycin selection. The cells were pelleted by centrifugation at 5.000× g for 10 min at 4°C and the cell pellets were stored at −80°C until further use.

### Purification of Tre-recombinase

Purification was carried out as previously described [28] with slight modifications. Briefly, the cells from an induced culture were harvested by centrifugation, resuspended in buffer 1 (50 mM Tris-HCl pH 8.0, 5 mM β-mercaptoethanol, 10% Glycerol, 1 M NaCl and 0.1% Tween 20) in the presence of “complete EDTA-free protease inhibitor cocktail” (Roche), and lysed by sonication. To the total lysate ice cold 1 M L-tartaric acid was added slowly and incubated for 5 min on ice. Insoluble debris was removed by centrifugation and subsequently the cleared lysate was filtered through 0.22 µm filter. The filtrate was directly loaded on to a 2 ml Ni-NTA beads column, pre-equilibrated with 20 mM Tris-HCl (pH 8.0) containing 300 mM NaCl. The column was washed with 100 ml of buffer 2 (20 mM Tris-HCl pH 8.0, 700 mM NaCl, 5% Glycerol, 0.1% Tween 20 and 20 mM Imidazole). Subsequently, the protein was eluted from the column in 5 ml of buffer 3 (20 mM Tris-HCl pH 8.0, 700 mM NaCl, 5% Glycerol, 5 mM β-mercaptoethanol and 250 mM Imidazole). The 5 ml fraction was filtered through 0.22 µm filter and directly loaded on S200 superdex column which was pre-equilibrated with buffer 4 (20 mM HEPES pH 7.4, 500 mM NaCl and 2 mM DTT) on ÄKTA purifier (GE Healthcare Life Sciences). The proteins were eluted as 1 ml fractions at 1 ml/min flow rate and purity of the protein in peak fractions was analyzed by 12% SDS-PAGE. Specificity of the purified proteins was confirmed by Western blotting using Tre-recombinase polyclonal antibodies.

### Cell culture and plasmids

HeLa (ATCC Cat# CCL-2) were maintained in Dulbecco's modified Eagle medium (DMEM) supplemented with 10% (v/v) fetal bovine serum (FBS), L-glutamine (2 mM), penicillin and streptomycin. Stable pSVLoxLTR HeLa cell lines were maintained in complete DMEM media with 3 µg/ml puromycin. CEM-SS cells (NIH AIDS Res. and Ref. Reagent Program Cat# 776) were cultivated in RPMI-1640 medium supplemented with 10% (v/v) FBS, L-glutamine (2 mM), penicillin and streptomycin. Transient transfections were carried out using TransIT (Mirus, USA) according to the instructions of the manufacturer. Plasmids pSVLoxLTR and p3Tre were used for the transient transfection recombination assays [16].

### Analysis of cellular toxicity

Cell viability was analyzed by measuring cellular metabolic activity using the alamarBlue redox indicator (Serotec), according to the manufacturer's protocol.

### Protein transduction of mammalian cells

Protein transduction was performed as reported previously [28] with slight modifications. The cells were seeded and grown overnight prior to transduction. The following day, the cell cultures were washed and incubated in serum free medium (optiMEM; Gibco) for 2 h at 37°C. The cells were then treated with recombinant Tre-recombinase proteins (1 µM) for 5 h in serum free medium on a rocker platform at 37°C, which was placed in the incubator. Subsequently, serum free media was replaced with complete growth media, containing penicillin and streptomycin, and further incubated for 48 h.

### Immunofluorescence analyses

HeLa cells were seeded onto 15 mm glass cover slips in 22 mm diameter dishes at a density of 1×10^5^ per well and grown overnight at 37°C. Protein transduction was carried out as described above. Five hours of post transduction the cells were washed three times with PBS, followed by washing with 0.5 mg/ml heparin in PBS (three times for 5 minutes each). For microscopic analysis, cells were fixed with 3% paraformaldehyde (PFA) for 20 min and then washed with 50 mM NH_4_Cl/PBS. The cells were permeabilized with 0.1% Triton X-100/PBS for 5 min and blocked with 0.5% BSA/PBS for 30 min at ambient temperature. Proteins were immunolabelled in 0.5% BSA/PBS using the Tre -recombinase polyclonal primary antibodies (1∶100), followed by FITC coupled secondary antibodies (Cy2; Molecular Probes Invitrogen). Nuclear DNA was visualized by Draq5 (Enzo Life Sciences). Samples were analyzed on a Axiovert 200 M microscope equipped with an LSM 510 META confocal laser scanning unit (Carl Zeiss) using a Plan-Apochromatic 63× oil immersion objective lens with a 1.4 numeric aperture. Image acquisition and processing was performed by using the Zeiss LSM imaging software.

### PCR analysis of Tre-recombination activity

In transient transfection experiments Tre recombination of LoxLTR sites was detected by PCR [16]. Briefly, 8–10×10^6^ HeLa were transiently transfected with 20 µg of the pSVLoxLTR reporter construct [16] using TransIT-LT1 (Mirus) according to the manufacturer's instructions. The following day, 2×10^5^ of the transfected cells were seeded in 35 mm diameter plates. In one culture, the expression plasmid p3Tre (1 µg) was co-transfected for positive control. The following day, protein transduction was carried out. At 48 hours post transduction, the cells were trypsinized, washed and genomic DNA was isolated using the Blood mini kit (Qiagen). For detection of the recombination product PCR was performed using 1 µg of genomic DNA as template and the P1 (5′-GCCTCGGCCTAGGAACAGT-3′) and P2 (5′-CCGCCACATATCCTGATCTT-3′) primer pairs. The amplification profile involved 30 cycles of denaturation at 94°C for 30 seconds, primer annealing at 52°C for 30 seconds and extension at 72°C for 2 min. Final extension was at 72°C for 10 minutes. PCR was performed in a total volume of 50 µl of 1×of 5-prime-PCR-mix (Thermo Scientific).

For internal control, expression plasmid-derived unrecombined *amp* sequences were detected as before using following oligonucleotide primer pairs: forward 5′-ATGAGTATTCAACATTTCCG-3′ and reverse 5′-TTACCAATGCTTAATCAGTGAGG-3′.

Detection of Tre recombination in stable pSVloxLTR HeLa reporter cells [16] was performed as follows. Protein transduction was carried out as before in a 10 cm diameter culture dish at a density of 1.2×10^6^ cells. After 48 h of protein transduction, whole genomic DNA was isolated from the transduced cells and PCR was performed using P1 and P2 specific primers to detect the recombination activity as described above. The p3Tre plasmid was transiently transfected as a positive control.

### Half-life determination of HTatNT and HTLMNT

HeLa cells (2**×**10^5^) were transduced with CPTR and their half-life was tested according to Peitz and coworkers [28]. After protein transduction, loosely bound proteins were removed by three washes with PBS followed by three washing steps (5 min each) with 0.5 mg per ml heparin in PBS. The cellular lysates were prepared for the indicated periods of time and the protein level in the lysates was detected by immunoblotting with anti-Tre polyclonal antibodies.

### Chromatin immunoprecipitation (ChIP) assay

ChIP assays were performed to determine the binding of CPTR to the specific LoxLTR sequence in living cells. HIV-1 infected CEM-SS cells were seeded with 2×10^6^ cells per well into 32 mm diameter culture dishes. HTLMNT and HTatNT proteins were transduced as described before and, at 3 hours post transduction, ChIP assay was performed using Tre-specific rabbit polyclonal antibody (5 µg) in combination with the Pierce Agarose ChIP Kit according to manufacturer's protocol (Thermo Scientific, Germany). After pull-down of specific DNA, PCR was performed using the LoxLTR-specific primers P3 (5′ TACTTCCCTGATTGGCAGAACTACACACC 3′) and P4 (5′ TCA AGA ACT GCT GAC ATC GAG C 3′). PCR products were subsequently visualized by agarose gel electrophoresis. The sequence was also confirmed by DNA sequencing.

### Excision of the full-length HIV-1 viral genome

HeLa and CEM-SS cell cultures were infected with HIV-1 pseudotypes, in which *env* sequences were deleted and the *nef* coding region was substituted by the *blasticidin* resistance gene, as described in detail previously [16]. Recombination activity of HTLMNT was determined as follows: 2×10^5^ infected HeLa cells or 2×10^6^ infected CEM-SS cells were seeded into 35 mm plates and grown overnight. The following day protein transduction was carried out as before and at 48 h post transduction whole genomic DNA was isolated for detection of the excised circular proviral DNA product by PCR using following primers: P5 forward primer 5′GCTGCCCTCTGGTTATGTGTG and P6 backward primer 5′ CTTAATACCGACGCTCTCGCAC.

## Supporting Information

Figure S1 **HTatNT Tat** ***trans*** **-activation analysis.** Analysis of *trans*-activation capacity of recombinant Tat and CPTR proteins.
(TIF)

Figure S2 **Effect of Tre-mediated recombination on Tat** ***trans*** **-activation.** Analysis of HTLMNT activity in transient Tat reporter assay.
(TIF)

Figure S3 **Gene expression analysis of CPTR-treated and untreated CEM-SS T cells.** Scatter plot of the changes in cellular gene expression in CPTR-treated vs. untreated cells.
(TIF)

Table S1 **Up-regulated genes in CPTR-treated vs. untreated CEM-SS T cells.**
(DOC)

Table S2 **Down-regulated genes in CPTR-treated vs. untreated CEM-SS T cells.**
(DOC)

File S1 **Supporting Methods and References.** Detailed legends of supporting figures and description of the related methods.
(DOC)
