# Supplementary figures and images for: Excision of HIV-1 Proviral DNA by Recombinant Cell Permeable Tre-Recombinase
Source: PLoS One. 2012 Feb 13;7(2):e31576. doi: 10.1371/journal.pone.0031576 (PMC3278460; doi:10.1371/journal.pone.0031576)

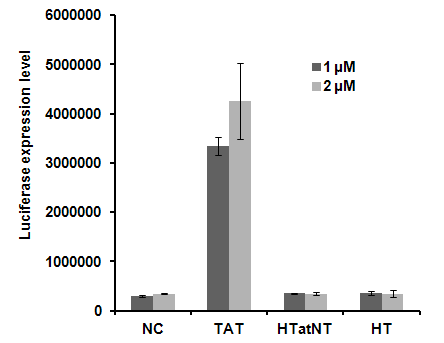

Supplement: Figure S1 — HTatNT Tat trans -activation analysis. Analysis of trans-activation capacity of recombinant Tat and CPTR proteins. (TIF) [file pone.0031576.s001.tif]

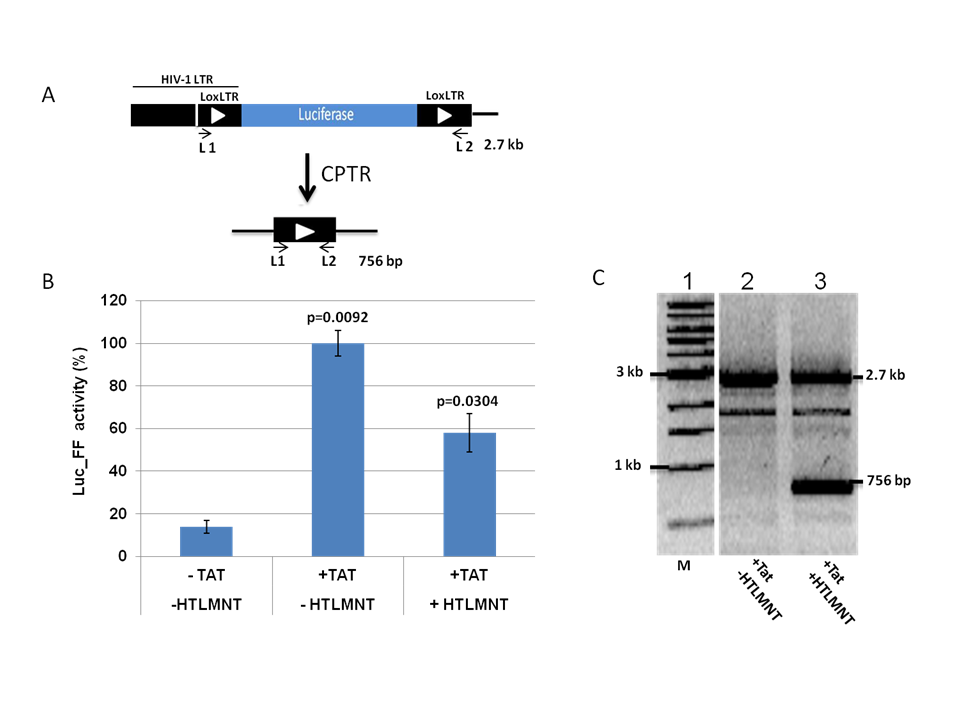

Supplement: Figure S2 — Effect of Tre-mediated recombination on Tat trans -activation. Analysis of HTLMNT activity in transient Tat reporter assay. (TIF) [file pone.0031576.s002.tif]

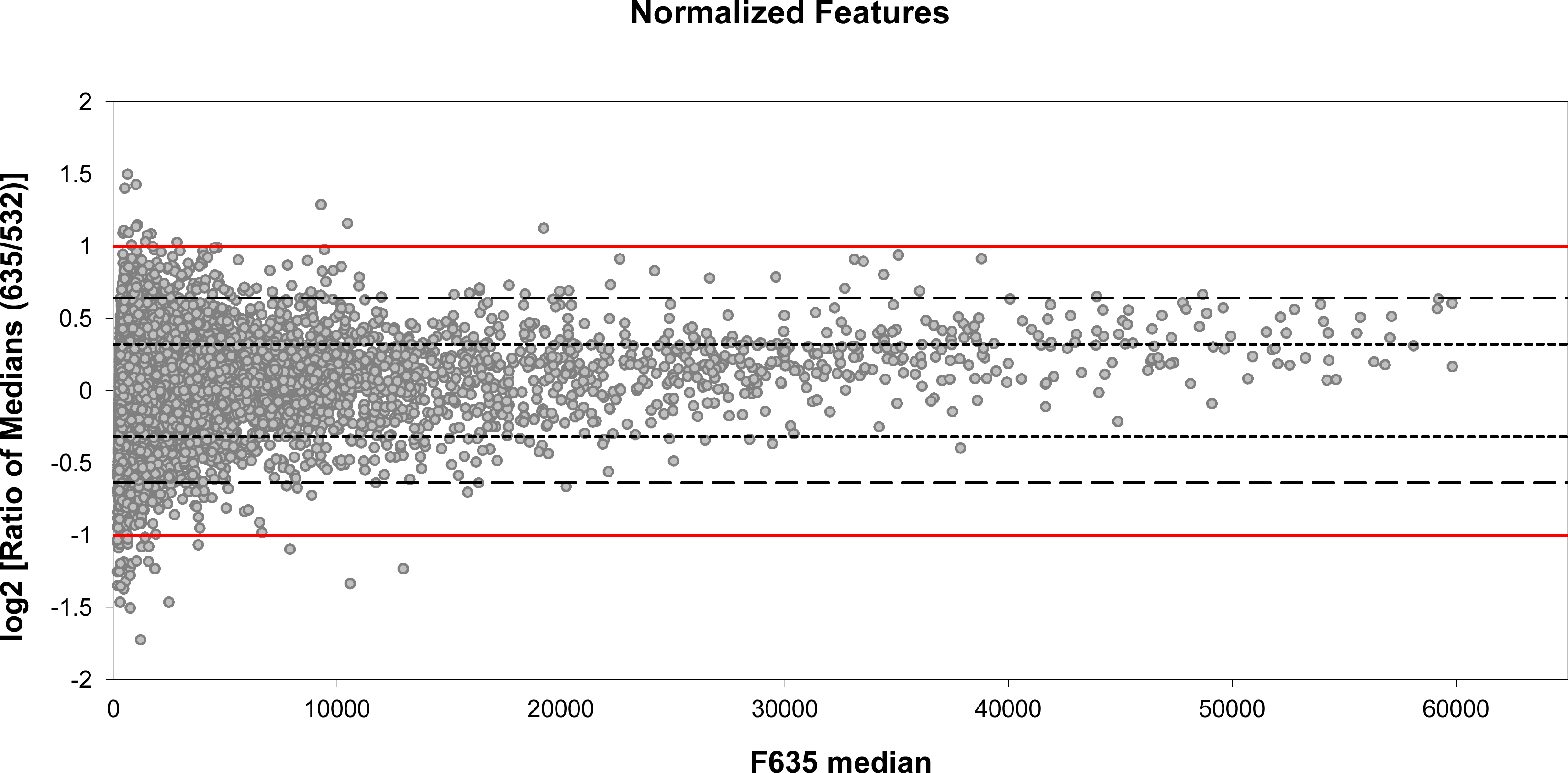

Supplement: Figure S3 — Gene expression analysis of CPTR-treated and untreated CEM-SS T cells. Scatter plot of the changes in cellular gene expression in CPTR-treated vs. untreated cells. (TIF) [file pone.0031576.s003.tif]
