# Supplementary material for: Excision of HIV-1 Proviral DNA by Recombinant Cell Permeable Tre-Recombinase
Source: PLoS One. 2012 Feb 13;7(2):e31576. doi: 10.1371/journal.pone.0031576 (PMC3278460; doi:10.1371/journal.pone.0031576)
Supplement: Table S1 — Up-regulated genes in CPTR-treated vs. untreated CEM-SS T cells. (DOC) [file pone.0031576.s004.doc]

**Table S1:** Up-regulated ^a^ genes in CPTR-treated vs. untreated CEM-SS cells.

**Fold-**

**Accession Change Gene Symbol Description**

NM_033510 2.83 DISP2 Homo sapiens dispatched homolog 2 (Drosophila) (DISP2), mRNA

NM_002165 2.69 ID1 Homo sapiens inhibitor of DNA binding 1, dominant negative helix- loop-helix protein (ID1), transcript variant 1, mRNA

NM_173653 2.65 SLC9A9 Homo sapiens solute carrier family 9 (sodium/hydrogen exchanger), member 9 (SLC9A9), mRNA

NM_005318 2.44 H1F0 Homo sapiens H1 histone family, member 0 (H1F0), mRNA

ENST00000283694 2.24 ENST00000283694 Homo sapiens Immunoglobulin variable region germ-line transcripts (RF.BM, YF.PB2 cell line).

NM_005655 2.22 KLF10 Homo sapiens Kruppel-like factor 10 (KLF10), transcript variant 1, mRNA

BC004179 2.20 MGC2780 Homo sapiens hypothetical protein MGC2780, mRNA (cDNA clone IMAGE:2959703)

NM_000421 2.18 KRT10 Homo sapiens keratin 10 (epidermolytic hyperkeratosis; keratosis palmaris et plantaris) (KRT10), mRNA

NM_002922 2.16 RGS1 Homo sapiens regulator of G-protein signalling 1 (RGS1), mRNA

NM_015948 2.14 SLC35B3 Homo sapiens solute carrier family 35, member B3 (SLC35B3), mRNA

NM_020734 2.14 FAM80B Homo sapiens family with sequence similarity 80, member B (FAM80B), mRNA

NM_016210 2.13 C3orf18 Homo sapiens chromosome 3 open reading frame 18 (C3orf18), mRNA

NM_004569 2.13 PIGH Homo sapiens phosphatidylinositol glycan anchor biosynthesis, class H (PIGH), mRNA

NM_005384 2.11 NFIL3 Homo sapiens nuclear factor, interleukin 3 regulated (NFIL3), mRNA

NM_006468 2.05 POLR3C Homo sapiens polymerase (RNA) III (DNA directed) polypeptide C (62kD) (POLR3C), mRNA

DQ926472 2.04 DQ926472 Homo sapiens clone IM2 3u45 immunoglobulin heavy chain variable region mRNA, partial cds.

AF035020 2.01 AF035020 Homo sapiens clone B2E9D5H myosin-reactive immunoglobulin heavy chain variable region mRNA, partial cds.

^a^ genes with more than 2fold up-regulation in CPTR-treated cells.
