# Supplementary material for: Excision of HIV-1 Proviral DNA by Recombinant Cell Permeable Tre-Recombinase
Source: PLoS One. 2012 Feb 13;7(2):e31576. doi: 10.1371/journal.pone.0031576 (PMC3278460; doi:10.1371/journal.pone.0031576)
Supplement: Table S2 — Down-regulated genes in CPTR-treated vs. untreated CEM-SS T cells. (DOC) [file pone.0031576.s005.doc]

**Table S2:** Down-regulated ^a^ genes in CPTR-treated vs. untreated CEM-SS cells.

**Fold-**

**Accession Change Gene Symbol Description**

NM_002130 -3.30 HMGCS1 Homo sapiens 3-hydroxy-3-methylglutaryl-Coenzyme A synthase 1 (soluble) (HMGCS1), mRNA

CN272797 -2.75 CN272797 CN272797 17000600009278 GRN_PREHEP Homo sapiens cDNA 5', mRNA sequence

CB250445 -2.75 CB250445 UI-CF-FN0-age-k-21-0-UI.s1 UI-CF-FN0 Homo sapiens cDNA clone UI-CF-FN0-age-k-21-0-UI 3', mRNA sequence

NM_001243 -2.54 TNFRSF8 Homo sapiens tumor necrosis factor receptor superfamily, member 8 (TNFRSF8), transcript variant 1, mRNA

NM_002574 -2.52 PRDX1 Homo sapiens peroxiredoxin 1 (PRDX1), transcript variant 1, mRNA

NM_006391 -2.49 IPO7 Homo sapiens importin 7 (IPO7), mRNA

ENST00000332148 -2.42 LOC441795 PREDICTED: Homo sapiens similar to high-mobility group box 3 (LOC441795), mRNA

NM_014509 -2.38 SERHL2 Homo sapiens serine hydrolase-like 2 (SERHL2), mRNA

NM_004454 -2.38 ETV5 Homo sapiens ets variant gene 5 (ets-related molecule) (ETV5),

mRNA

NM_000527 -2.35 LDLR Homo sapiens low density lipoprotein receptor (familial hypercholesterolemia) (LDLR), mRNA

NM_006597 -2.35 HSPA8 Homo sapiens heat shock 70kDa protein 8 (HSPA8), transcript variant 1, mRNA

NM_014762 -2.34 DHCR24 Homo sapiens 24-dehydrocholesterol reductase (DHCR24), mRNA

XR_019230 -2.29 LOC442210 PREDICTED: Homo sapiens similar to tubulin, beta 5 (LOC442210), mRNA

AI608782 -2.29 AI608782 AI608782 tw94g05.x1 NCI_CGAP_HN6 Homo sapiens cDNA clone IMAGE:2267384 3' similar to gb:K00558 TUBULIN ALPHA-1 CHAIN (HUMAN);, mRNA sequence

NM_001456 -2.28 FLNA Homo sapiens filamin A, alpha (actin binding protein 280) (FLNA), mRNA

THC2598478 -2.27 THC2598478 MUSHSP84B heat-shock protein hsp84 {Mus musculus} (exp=-1; wgp=0; cg=0), partial (17%)

NM_178014 -2.14 TUBB Homo sapiens tubulin, beta (TUBB), mRNA

AL512694 -2.12 AL512694 Homo sapiens mRNA; cDNA DKFZp761J2423 (from clone DKFZp761J2423).

XR_018043 -2.11 LOC442308 PREDICTED: Homo sapiens similar to tubulin, beta 5 (LOC442308), mRNA

XR_018797 -2.08 LOC645360 PREDICTED: Homo sapiens similar to high-mobility group box 3 (LOC645360), mRNA

NM_024830 -2.08 AYTL2 Homo sapiens acyltransferase like 2 (AYTL2), mRNA

NM_001018111 -2.07 PODXL Homo sapiens podocalyxin-like (PODXL), transcript variant 1, mRNA

CR598370 -2.04 CR598370 full-length cDNA clone CS0DF003YI18 of Fetal brain of Homo sapiens (human).

AF132203 -2.04 AF132203 Homo sapiens PRO1933 mRNA, complete cds.

NM_006805 -2.02 HNRPA0 Homo sapiens heterogeneous nuclear ribonucleoprotein A0 (HNRPA0), mRNA

^a^ genes with more than 2fold repression in CPTR-treated cells.
