# Supplementary material for: Excision of HIV-1 Proviral DNA by Recombinant Cell Permeable Tre-Recombinase
Source: PLoS One. 2012 Feb 13;7(2):e31576. doi: 10.1371/journal.pone.0031576 (PMC3278460; doi:10.1371/journal.pone.0031576)
Supplement: File S1 — Supporting Methods and References. Detailed legends of supporting figures and description of the related methods. (DOC) [file pone.0031576.s006.doc]

**Supporting Material**

**Excision of HIV-1 Proviral DNA by Recombinant Cell Permeable Tre-Recombinase**

Lakshmikanth Mariyanna, Poornima Priyadarshini, Helga Hofmann-Sieber, Marcel Krepstakies, Nicole Walz, Adam Grundhoff, Frank Buchholz, Eberhard Hildt and Joachim Hauber

**Methods**

**Expression analysis using whole human genome microarrays**

Labeling of 1 µg control and sample RNA was performed using the Quick Amp Labeling Kit (Agilent, two-color, 5190-0444) according to the manufacturers’ recommendations, and array hybridization and quality control was performed as described in the Agilent Two-Color Microarray-Based Gene Expression Analysis protocol (Version 5.0.1, August 2006).

Briefly, quality and integrity of total input RNA was assessed using the 2100 Bioanalyzer and the RNA 6000 Nano Assay Kit (Agilent Technologies). Control RNA and sample RNA including spike in controls were transcribed into cDNA using a T7 promotor containing primer. Subsequently the cDNA served as the template for cRNA synthesis with T7 RNA Polymerase. During this step Cyanine-3 or Cyanine-5 labeled CTP were incorporated into control or sample RNA, respectively. RNA was purified with the RNeasy Mini Kit (Qiagen) and measured for proper dye incorporation with the NanoDrop ND-1000 (peqLab). Equal amounts of control and sample RNA (825 ng each) were hybridized competitively to a 44K Whole Human Genome Oligo Microarray (Agilent, G4112F) in a rotating oven at 10 rpm for 17 h at 65°C. After disassembly, the microarray was washed for 1 min in Gene Expression Wash Buffer 1 (Agilent, 5188-5325) at room temperature and for 1 min in pre-warmed (37°C) Gene Expression Wash Buffer 2 (Agilent, 5188-5326), dried shortly in a slide centrifuge and scanned using a GenePix Personal 4100A scanner (Axon Instruments).

Analysis and normalization was performed using the GenePix Pro 6.0 software (Axon Instruments). Briefly, the DNA gene expression array grid template was adjusted to the array and subsequently all features were aligned and analyzed. Integrated settings were further used to flag features as “good” when fulfilling the “fair feature” criteria (i.e. (i) at least 55% of a spots pixels must be above the mean plus a single standard deviation unit of the background fluorescence value, (ii) less than 3% of the spot's pixels may be saturated, and (iii) the sum of median pixel fluorescence intensity of both channels must be above 500). For normalization purposes, all spots flagged as “good” were included, whereas control spots were excluded. Normalization was performed such that the average ratio of medians of all normalization features is equal to 1. A twofold change in the expression level was selected as a cut-off to determine differentially expressed genes as shown in Tables S1 and S2.

**Tat *trans*-activation assays**

Tat *trans*-activation was investigated using the reporter plasmid pHIV/T2/LUC [1] in the presence or absence of Tat and Tre protein (i.e. HTLMNT). A culture of 3 x 10^5^ HeLa cells were transfected with 2.25 μg of pHIV/T2/LUC DNA. Post transfection the cells were split and seeded into three separate 32 mm diameter culture dishes (1 x 10^5^ cells each). Subsequently, the respective cultures were transfected with 250 ng of either pcTat expression plasmid [2], or the negative control vector pcDNA3 (Invitrogen). The following day, cells were transduced with HTLMNT protein. Cellular lysates were prepared 48 hours after transduction for analysis of luciferase expression using the luciferase assay system (Promega) according to manufacturer’s protocol. A parallel experimental set-up was used for isolation of total genomic DNA for PCR analysis.

For analysis of the potential Tat *trans*-activation capacity of HTatNT protein, TZM-bl HeLa Tat reporter cells (NIH AIDS Res. and Ref. Reagent Program Cat# 8129) [3,4], harboring the luciferase gene under the control of the HIV-1 LTR promoter, were seeded into 96 well dishes (1 x 10^4^ cells per well). Full length recombinant Tat protein (positive control; Cat#: ab83353, Abcam), HTatNT and HT (negative control protein) were transduced as described before and luciferase activity was measured 48 hours post protein transduction.

**Supplemental Figure Legends**

**Figure S1. HTatNT Tat *trans*-activation analysis.** TZM-bl Tat reporter cells were transduced with the indicated purified proteins using a protein concentration of 1 µM and 2 µM. Luciferase activity was measured at 48 hours post transduction. NC, negative control (untreated cells).

**Figure S2. Effect of Tre-mediated recombination on Tat *trans*-activation.** (A) Schematic presentation of the HIV-1 Tat responsive reporter vector pHIV/T2/Luc, which is characterized by an HIV-1 LTR, containing a LoxLTR sequence within its U3 region, and another LoxLTR sequence located 3’ of the luciferase reporter gene. L1 and L2 denote the primer pair used for PCR detection of recombination (forward and reverse arrows). The size of the PCR products before and after recombination are indicated. (B) Effect of Tre on Tat *trans*-activation. HeLa cells were cotransfected with the Tat responsive reporter plasmid pHIV/T2/LUC, a Tat-expressing vector, or the respective parental plasmid (Tat-deficient control). Subsequently, protein transduction was carried out with HTLMNT and the relative HIV-1 LTR activity in presence and absence of Tre was analyzed by measuring luciferase activity. Numbers over the bars indicate the p-values determined by paired t-test. At 48 hours post HTLMNT transduction, Tat activity was decreased by 40%. (C) Isolation of total genomic DNA at 48 hours post protein transduction. PCR analysis of recombination was performed by using the L1 and L2 primer pair.

**Figure S3: Gene expression analysis of CPTR-treated and untreated CEM-SS T cells.** Shown is a scatter plot of the expression changes (log2 ratio of median spot intensity) in CPTR-treated vs. untreated cells (F635 and F532 channel, respectively), plotted against the expression levels (median spot intensity) in the CPTR-treated samples. The one- and two-fold standard deviation values of expression changes across all features are marked by dotted and dashed lines, respectively. The cutoff value for 2fold up- or down-regulated features (log2 ratio of 1 and -1, respectively) is marked by a solid red line.

**Supplemental Tables**

**Table S1:** Up-regulated genes in CPTR-treated vs. untreated CEM-SS T cells.

**Table S2:** Down-regulated genes in CPTR-treated vs. untreated CEM-SS T cells.

**Supplemental References**

1. Sarkar I, Hauber I, Hauber J, Buchholz F (2007) HIV-1 proviral DNA excision using an evolved recombinase. Science 316: 1912-1915.

2. Malim MH, Hauber J, Fenrick R, Cullen BR (1988) Immunodeficiency virus rev trans-activator modulates the expression of the viral regulatory genes. Nature 335: 181-183.

3. Wei X, Decker JM, Liu H, Zhang Z, Arani RB et al. (2002) Emergence of resistant human immunodeficiency virus type 1 in patients receiving fusion inhibitor (T-20) monotherapy. Antimicrob.Agents Chemother. 46: 1896-1905.

4. Derdeyn CA, Decker JM, Sfakianos JN, Wu X, O'Brien WA et al. (2000) Sensitivity of human immunodeficiency virus type 1 to the fusion inhibitor T-20 is modulated by coreceptor specificity defined by the V3 loop of gp120. J.Virol. 74: 8358-8367.
